# Supplementary material for: Meta-analysis of commonly mutated genes in leptomeningeal carcinomatosis
Source: PeerJ. 2023 Apr 19;11:e15250. doi: 10.7717/peerj.15250 (PMC10122459; doi:10.7717/peerj.15250)

GO:BP		stats						
Term name	Term ID	p_{adj}	$-\log_{10}(p_{adj})$ 0 ≤16	TP53	PIK3CA	KMT2D	PTEN	IL7R
regulation of lymphocyte apoptotic process	GO:0070228	1.574×10^{-4}						
lymphocyte apoptotic process	GO:0070227	3.727×10^{-4}						
regulation of fibroblast apoptotic process	GO:2000269	4.882×10^{-4}						
regulation of leukocyte apoptotic process	GO:2000106	5.210×10^{-4}						
developmental growth	GO:0048589	6.375×10^{-4}						
fibroblast apoptotic process	GO:0044346	6.820×10^{-4}						
leukocyte apoptotic process	GO:0071887	1.158×10^{-3}						
positive regulation of signal transduction	GO:0009967	1.432×10^{-3}						
negative regulation of macroautophagy	GO:0016242	2.136×10^{-3}						
regulation of cell population proliferation	GO:0042127	2.333×10^{-3}						
positive regulation of cell communication	GO:0010647	2.638×10^{-3}						
positive regulation of signaling	GO:0023056	2.670×10^{-3}						
growth	GO:0040007	3.300×10^{-3}						
cell population proliferation	GO:0008283	5.279×10^{-3}						
positive regulation of response to stimulus	GO:0048584	8.056×10^{-3}						
peptidyl-amino acid modification	GO:0018193	1.041×10^{-2}						
cellular response to environmental stimulus	GO:0104004	1.075×10^{-2}						
cellular response to abiotic stimulus	GO:0071214	1.075×10^{-2}						
negative regulation of apoptotic process	GO:0043066	1.097×10^{-2}						
negative regulation of programmed cell death	GO:0043069	1.185×10^{-2}						
negative regulation of autophagy	GO:0010507	1.251×10^{-2}						
positive regulation of cell population proliferation	GO:0008284	1.425×10^{-2}						
negative regulation of cell death	GO:0060548	1.727×10^{-2}						
chromatin organization	GO:0006325	1.816×10^{-2}						
circadian behavior	GO:0048512	1.940×10^{-2}						
rhythmic behavior	GO:0007622	2.126×10^{-2}						
regulation of T cell apoptotic process	GO:0070232	2.254×10^{-2}						
regulation of cell aging	GO:0090342	2.422×10^{-2}						
regulation of generation of precursor metabolites and e...	GO:0043467	3.057×10^{-2}						
regulation of cellular component size	GO:0032535	3.692×10^{-2}						
regulation of DNA metabolic process	GO:0051052	3.723×10^{-2}						
negative regulation of pentose-phosphate shunt	GO:1905856	3.774×10^{-2}						
negative regulation of G1 to G0 transition	GO:1903451	3.774×10^{-2}						
regulation of transcription from RNA polymerase II prom...	GO:1990248	3.774×10^{-2}						
multicellular organism growth	GO:0035264	3.958×10^{-2}						
regulation of macroautophagy	GO:0016241	4.065×10^{-2}						
regulation of signal transduction	GO:0009966	4.148×10^{-2}						
cellular response to chemical stimulus	GO:0070887	4.739×10^{-2}						
T cell apoptotic process	GO:0070231	4.922×10^{-2}						

1 to 39 of 39 | Page 1 of 1

REAC		stats						
Term name	Term ID	p_{adj}	$-\log_{10}(p_{adj})$ 0 ≤16	TP53	PIK3CA	KMT2D	PTEN	IL7R
PIP3 activates AKT signaling	REAC:R-HSA-1...	9.140×10^{-3}						
Ovarian tumor domain proteases	REAC:R-HSA-5...	1.329×10^{-2}						
Intracellular signaling by second messengers	REAC:R-HSA-9...	1.420×10^{-2}						
Synthesis of PIPs at the plasma membrane	REAC:R-HSA-1...	2.502×10^{-2}						
Regulation of TP53 Expression	REAC:R-HSA-6...	3.404×10^{-2}						

1 to 5 of 5 | Page 1 of 1

The colors for different evidence codes in the table:

Gene Ontology
- Inferred from experiment [IDA, IPI, IMP, IGI, IEP]
- Direct assay [IDA], Mutant phenotype [IMP]
- Genetic interaction [IGI], Physical interaction [IPI]
- Inferred from High Throughput Experiment [HDA, HMP, HGI, HEP]
- High Throughput Direct Assay [HDA], High Throughput Mutant Phenotype [HMP]
- High Throughput Genetic interaction [HGI], High Throughput Expression pattern [HEP]
- Traceable author [TAS], Non-traceable author [NAS], Inferred by curator [IC]
- Expression pattern [IEP], Sequence or structural similarity [ISS], Genomic context [IGC]
- Sequence Model [ISM], Sequence Alignment [ISA], Sequence Orthology [ISO]
- Biological aspect of ancestor [IBA], Rapid divergence [IRD]
- Reviewed computational analysis [RCA], Electronic annotation [IEA]
- No biological data [ND], Not annotated or not in background [NA]

Biological pathways
- KEGG , Reactome

Regulatory motifs in DNA
- TRANSFAC TFBS , miRTarBase

Human Protein Atlas
- [Enhanced, Supported, Approved]

CORUM protein complexes
- CORUM

Human Phenotype Ontology
- Human Phenotype Ontology (sequence homologs in other species)

The colors for log scale:

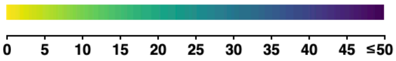

Supplement: Supplemental Information 1 — Pathway enrichment analysis of commonly mutated genes in NSCLC, Breast, Melanoma LMC patients using g:Profiler (https://biit.cs.ut.ee/gprofiler/gost) [file peerj-11-15250-s001.pdf]
