# Supplementary material for: Meta-analysis of commonly mutated genes in leptomeningeal carcinomatosis
Source: PeerJ. 2023 Apr 19;11:e15250. doi: 10.7717/peerj.15250 (PMC10122459; doi:10.7717/peerj.15250)
Supplement: Supplemental Information 2 [file peerj-11-15250-s002.docx]

**PubMed**

**Keywords:** (Leptomeningeal carcinomatosis OR leptomeningeal carcinoma OR leptomeningeal disease OR leptomeningeal metastases OR leptomeningeal metastasis) AND (liquid biopsy OR cerebrospinal fluid OR cerebral spinal fluid OR circulating tumor cells) AND (Next-generation sequencing OR RNA sequencing OR whole exome sequencing OR mutation OR non-small cell lung cancer OR NSCLC OR breast cancer OR melanoma) NOT (review OR drug trial OR clinical triall)

Filters: English

((("meningeal carcinomatosis"[MeSH Terms] OR ("meningeal"[All Fields] AND "carcinomatosis"[All Fields]) OR "meningeal carcinomatosis"[All Fields] OR ("leptomeningeal"[All Fields] AND "carcinomatosis"[All Fields]) OR "leptomeningeal carcinomatosis"[All Fields] OR (("leptomeninge"[All Fields] OR "leptomeningeal"[All Fields] OR "leptomeninges"[All Fields] OR "leptomeningitis"[All Fields]) AND ("carcinoma"[MeSH Terms] OR "carcinoma"[All Fields] OR "carcinomas"[All Fields] OR "carcinoma s"[All Fields])) OR (("leptomeninge"[All Fields] OR "leptomeningeal"[All Fields] OR "leptomeninges"[All Fields] OR "leptomeningitis"[All Fields]) AND ("disease"[MeSH Terms] OR "disease"[All Fields] OR "diseases"[All Fields] OR "disease s"[All Fields] OR "diseased"[All Fields])) OR (("leptomeninge"[All Fields] OR "leptomeningeal"[All Fields] OR "leptomeninges"[All Fields] OR "leptomeningitis"[All Fields]) AND ("metastasation"[All Fields] OR "metastasic"[All Fields] OR "metastasing"[All Fields] OR "metastasise"[All Fields] OR "metastasised"[All Fields] OR "metastasises"[All Fields] OR "metastasising"[All Fields] OR "metastasization"[All Fields] OR "metastasizes"[All Fields] OR "metastasizing"[All Fields] OR "neoplasm metastasis"[MeSH Terms] OR ("neoplasm"[All Fields] AND "metastasis"[All Fields]) OR "neoplasm metastasis"[All Fields] OR "metastase"[All Fields] OR "metastases"[All Fields] OR "metastasize"[All Fields] OR "metastasized"[All Fields])) OR (("leptomeninge"[All Fields] OR "leptomeningeal"[All Fields] OR "leptomeninges"[All Fields] OR "leptomeningitis"[All Fields]) AND ("metastasi"[All Fields] OR "neoplasm metastasis"[MeSH Terms] OR ("neoplasm"[All Fields] AND "metastasis"[All Fields]) OR "neoplasm metastasis"[All Fields] OR "metastasis"[All Fields]))) AND ("liquid biopsy"[MeSH Terms] OR ("liquid"[All Fields] AND "biopsy"[All Fields]) OR "liquid biopsy"[All Fields] OR ("cerebrospinal fluid"[MeSH Subheading] OR ("cerebrospinal"[All Fields] AND "fluid"[All Fields]) OR "cerebrospinal fluid"[All Fields] OR "cerebrospinal fluid"[MeSH Terms] OR ("cerebrospinal"[All Fields] AND "fluid"[All Fields])) OR ("cerebrospinal fluid"[MeSH Terms] OR ("cerebrospinal"[All Fields] AND "fluid"[All Fields]) OR "cerebrospinal fluid"[All Fields] OR ("cerebral"[All Fields] AND "spinal"[All Fields] AND "fluid"[All Fields]) OR "cerebral spinal fluid"[All Fields]) OR ("neoplastic cells, circulating"[MeSH Terms] OR ("neoplastic"[All Fields] AND "cells"[All Fields] AND "circulating"[All Fields]) OR "circulating neoplastic cells"[All Fields] OR ("circulating"[All Fields] AND "tumor"[All Fields] AND "cells"[All Fields]) OR "circulating tumor cells"[All Fields])) AND ("high throughput nucleotide sequencing"[MeSH Terms] OR ("high throughput"[All Fields] AND "nucleotide"[All Fields] AND "sequencing"[All Fields]) OR "high throughput nucleotide sequencing"[All Fields] OR ("next"[All Fields] AND "generation"[All Fields] AND "sequencing"[All Fields]) OR "next generation sequencing"[All Fields] OR ("sequence analysis, rna"[MeSH Terms] OR ("sequence"[All Fields] AND "analysis"[All Fields] AND "rna"[All Fields]) OR "rna sequence analysis"[All Fields] OR ("rna"[All Fields] AND "sequencing"[All Fields]) OR "rna sequencing"[All Fields]) OR ("exome sequencing"[MeSH Terms] OR ("exome"[All Fields] AND "sequencing"[All Fields]) OR "exome sequencing"[All Fields] OR ("whole"[All Fields] AND "exome"[All Fields] AND "sequencing"[All Fields]) OR "whole exome sequencing"[All Fields]) OR ("mutate"[All Fields] OR "mutated"[All Fields] OR "mutates"[All Fields] OR "mutating"[All Fields] OR "mutation"[MeSH Terms] OR "mutation"[All Fields] OR "mutations"[All Fields] OR "mutation s"[All Fields] OR "mutational"[All Fields] OR "mutator"[All Fields] OR "mutators"[All Fields]) OR ("carcinoma, non small cell lung"[MeSH Terms] OR ("carcinoma"[All Fields] AND "non small cell"[All Fields] AND "lung"[All Fields]) OR "non-small-cell lung carcinoma"[All Fields] OR ("non"[All Fields] AND "small"[All Fields] AND "cell"[All Fields] AND "lung"[All Fields] AND "cancer"[All Fields]) OR "non small cell lung cancer"[All Fields]) OR ("carcinoma, non small cell lung"[MeSH Terms] OR ("carcinoma"[All Fields] AND "non small cell"[All Fields] AND "lung"[All Fields]) OR "non-small-cell lung carcinoma"[All Fields] OR "nsclc"[All Fields] OR "nsclc s"[All Fields] OR "nsclcs"[All Fields]) OR ("breast neoplasms"[MeSH Terms] OR ("breast"[All Fields] AND "neoplasms"[All Fields]) OR "breast neoplasms"[All Fields] OR ("breast"[All Fields] AND "cancer"[All Fields]) OR "breast cancer"[All Fields]) OR ("melanoma"[MeSH Terms] OR "melanoma"[All Fields] OR "melanomas"[All Fields] OR "melanoma s"[All Fields]))) NOT ("review"[Publication Type] OR "review literature as topic"[MeSH Terms] OR "review"[All Fields] OR ("drug"[All Fields] AND ("clinical trials as topic"[MeSH Terms] OR ("clinical"[All Fields] AND "trials"[All Fields] AND "topic"[All Fields]) OR "clinical trials as topic"[All Fields] OR "trial"[All Fields] OR "trial s"[All Fields] OR "trialed"[All Fields] OR "trialing"[All Fields] OR "trials"[All Fields])) OR (("ambulatory care facilities"[MeSH Terms] OR ("ambulatory"[All Fields] AND "care"[All Fields] AND "facilities"[All Fields]) OR "ambulatory care facilities"[All Fields] OR "clinic"[All Fields] OR "clinic s"[All Fields] OR "clinical"[All Fields] OR "clinically"[All Fields] OR "clinicals"[All Fields] OR "clinics"[All Fields]) AND "triall"[All Fields]))) AND (english[Filter]))
