# Supplementary material for: Meta-analysis of commonly mutated genes in leptomeningeal carcinomatosis
Source: PeerJ. 2023 Apr 19;11:e15250. doi: 10.7717/peerj.15250 (PMC10122459; doi:10.7717/peerj.15250)
Supplement: Supplemental Information 4 [file peerj-11-15250-s004.docx]

1. The rationale for conducting the meta-analysis.

Limited number of studies (Boire et al. 2017; Chi et al. 2020; Fan et al. 2018; Li et al. 2021; Ruan et al. 2020; Smalley et al. 2020; Smalley et al. 2021) investigated the differentially expressed genes (DEGs) and used multi-omics data to understand the LMC progression. Only two meta-analyses (Tewarie et al. 2021; Wang et al. 2022) were conducted to analyze potential diagnostic values and assess risk factors for LMC. Nevertheless, one of the main challenges is that these studies focused on different primary tumor sites and different aspects in LMC progression such as DEGs, tumor microenvironment, or immune landscape. Therefore, this study aims to identify possible molecular pathways that may be involved in LMC development.

1. The contribution that the meta-analysis makes to knowledge in light of previously published related reports, including other meta-analyses and systematic reviews.

This study fills the gap in the literature by conducting a meta-analysis by gathering the information of mutated genes from a total of 16 articles about LMC caused by NSCLC, breast cancer, and melanoma. The outcome enables the identification of commonly mutated genes in three primary cancers, sheds light into the molecular mechanism of LMC, and finally finds possible targets for therapy.

Boire A, Zou Y, Shieh J, Macalinao DG, Pentsova E, Massagué J. Complement Component 3 Adapts the Cerebrospinal Fluid for Leptomeningeal Metastasis. Cell. 2017 Mar 9;168(6):1101-1113.e13. doi: 10.1016/j.cell.2017.02.025. PMID: 28283064; PMCID: PMC5405733.

Chi Y, Remsik J, Kiseliovas V, Derderian C, Sener U, Alghader M, Saadeh F, Nikishina K, Bale T, Iacobuzio-Donahue C, Thomas T, Pe'er D, Mazutis L, Boire A. Cancer cells deploy lipocalin-2 to collect limiting iron in leptomeningeal metastasis. Science. 2020 Jul 17;369(6501):276-282. doi: 10.1126/science.aaz2193. PMID: 32675368; PMCID: PMC7816199.

Fan Y, Zhu X, Xu Y, Lu X, Xu Y, Wang M, Xu H, Ding J, Ye X, Fang L, Huang Z, Gong L, Lu H, Mao W, Hu M. Cell-Cycle and DNA-Damage Response Pathway Is Involved in Leptomeningeal Metastasis of Non-Small Cell Lung Cancer. Clin Cancer Res. 2018 Jan 1;24(1):209-216. doi: 10.1158/1078-0432.CCR-17-1582. Epub 2017 Oct 13. PMID: 29030356.

​​Li Y, Polyak D, Lamsam L, Connolly ID, Johnson E, Khoeur LK, Andersen S, Granucci M, Stanley G, Liu B, Nagpal S, Hayden Gephart M. Comprehensive RNA analysis of CSF reveals a role for CEACAM6 in lung cancer leptomeningeal metastases. NPJ Precis Oncol. 2021 Oct 8;5(1):90. doi: 10.1038/s41698-021-00228-6. PMID: 34625644; PMCID: PMC8501028.

Ruan H, Zhou Y, Shen J, Zhai Y, Xu Y, Pi L, Huang R, Chen K, Li X, Ma W, Wu Z, Deng X, Wang X, Zhang C, Guan M. Circulating tumor cell characterization of lung cancer brain metastases in the cerebrospinal fluid through single-cell transcriptome analysis. Clin Transl Med. 2020 Dec;10(8):e246. doi: 10.1002/ctm2.246. PMID: 33377642; PMCID: PMC7737787.

Smalley I, Chen Z, Phadke M, Li J, Yu X, Wyatt C, Evernden B, Messina JL, Sarnaik A, Sondak VK, Zhang C, Law V, Tran N, Etame A, Macaulay RJB, Eroglu Z, Forsyth PA, Rodriguez PC, Chen YA, Smalley KSM. Single-Cell Characterization of the Immune Microenvironment of Melanoma Brain and Leptomeningeal Metastases. Clin Cancer Res. 2021 Jul 15;27(14):4109-4125. doi: 10.1158/1078-0432.CCR-21-1694. Epub 2021 May 25. PMID: 34035069; PMCID: PMC8282775.

Smalley I, Law V, Wyatt C, Evernden B, Fang B, Koomen JM, Welsh EA, Macaulay RJB, Forsyth PA, Smalley KSM. Proteomic Analysis of CSF from Patients with Leptomeningeal Melanoma Metastases Identifies Signatures Associated with Disease Progression and Therapeutic Resistance. Clin Cancer Res. 2020 May 1;26(9):2163-2175. doi: 10.1158/1078-0432.CCR-19-2840. Epub 2020 Jan 10. PMID: 31924735; PMCID: PMC7196498.

Tewarie IA, Jessurun CAC, Hulsbergen AFC, Smith TR, Mekary RA, Broekman MLD. Leptomeningeal disease in neurosurgical brain metastases patients: A systematic review and meta-analysis. Neurooncol Adv. 2021 Nov 10;3(1):vdab162. doi: 10.1093/noajnl/vdab162. PMID: 34859226; PMCID: PMC8633671.

Wang H, Wang L, Fang C, Li C, Zhang L. Comparison of the diagnostic value of liquid biopsy in leptomeningeal metastases: A systematic review and meta-analysis. Front Oncol. 2022 Dec 19;12:1079796. doi: 10.3389/fonc.2022.1079796. PMID: 36601482; PMCID: PMC9806138.
