# Supplementary material for: Meta-analysis of commonly mutated genes in leptomeningeal carcinomatosis
Source: PeerJ. 2023 Apr 19;11:e15250. doi: 10.7717/peerj.15250 (PMC10122459; doi:10.7717/peerj.15250)
Supplement: Supplemental Information 6 [file peerj-11-15250-s006.docx]

**Supplementary Table 1.** Excluded studies

| **Study** | **Author** | **Reason** |
| --- | --- | --- |
| Comprehensive RNA analysis of CSF reveals a role for CEACAM6 in lung cancer leptomeningeal metastases | Li et al. 2021 | Not enough mutation data |
| Detection of Leptomeningeal Disease Using Cell-Free DNA From Cerebrospinal Fluid | White et al. 2021 | Not enough mutation data |
| Tumor DNA Mutations From Intraparenchymal Brain Metastases Are Detectable in CSF | Cheok et al. 2021 | Not enough mutation data |
| Cancer cells deploy lipocalin-2 to collect limiting iron in leptomeningeal metastasis | Chi et al. 2020 | Not enough mutation data |
| Circulating Tumor DNA Analysis for Patients with Oncogene-Addicted NSCLC With Isolated Central Nervous System Progression | Aldea et al. 2020 | Tumor cells not derived from CFS |
| Biopsy-free circulating tumor DNA assay identifies actionable mutations in lung cancer | Villaflor et al. 2016 | Tumor cells not derived from CFS |

CSF, cerebrospinal fluid

Aldea M, Hendriks L, Mezquita L, Jovelet C, Planchard D, Auclin E, Remon J, Howarth K, Benitez JC, Gazzah A, Lavaud P, Naltet C, Lacroix L, de Kievit F, Morris C, Green E, Ngo-Camus M, Rouleau E, Massard C, Caramella C, Friboulet L, Besse B. Circulating Tumor DNA Analysis for Patients with Oncogene-Addicted NSCLC With Isolated Central Nervous System Progression. J Thorac Oncol. 2020 Mar;15(3):383-391. doi: 10.1016/j.jtho.2019.11.024. Epub 2019 Dec 13. PMID: 31843682.

Cheok SK, Narayan A, Arnal-Estape A, Gettinger S, Goldberg SB, Kluger HM, Nguyen D, Patel A, Chiang V. Tumor DNA Mutations From Intraparenchymal Brain Metastases Are Detectable in CSF. JCO Precis Oncol. 2021 Jan 12;5:PO.20.00292. doi: 10.1200/PO.20.00292. PMID: 34250381; PMCID: PMC8232069.

Chi Y, Remsik J, Kiseliovas V, Derderian C, Sener U, Alghader M, Saadeh F, Nikishina K, Bale T, Iacobuzio-Donahue C, Thomas T, Pe'er D, Mazutis L, Boire A. Cancer cells deploy lipocalin-2 to collect limiting iron in leptomeningeal metastasis. Science. 2020 Jul 17;369(6501):276-282. doi: 10.1126/science.aaz2193. PMID: 32675368; PMCID: PMC7816199.

Li Y, Polyak D, Lamsam L, Connolly ID, Johnson E, Khoeur LK, Andersen S, Granucci M, Stanley G, Liu B, Nagpal S, Hayden Gephart M. Comprehensive RNA analysis of CSF reveals a role for CEACAM6 in lung cancer leptomeningeal metastases. NPJ Precis Oncol. 2021 Oct 8;5(1):90. doi: 10.1038/s41698-021-00228-6. PMID: 34625644; PMCID: PMC8501028.

Villaflor V, Won B, Nagy R, Banks K, Lanman RB, Talasaz A, Salgia R. Biopsy-free circulating tumor DNA assay identifies actionable mutations in lung cancer. Oncotarget. 2016 Oct 11;7(41):66880-66891. doi: 10.18632/oncotarget.11801. PMID: 27602770; PMCID: PMC5341844.

White MD, Klein RH, Shaw B, Kim A, Subramanian M, Mora JL, Giobbie-Hurder A, Nagabhushan D, Jain A, Singh M, Kuter BM, Nayyar N, Bertalan MS, Stocking JH, Markson SC, Lastrapes M, Alvarez-Breckenridge C, Cahill DP, Gydush G, Rhoades J, Rotem D, Adalsteinsson VA, Mahar M, Kaplan A, Oh K, Sullivan RJ, Gerstner E, Carter SL, Brastianos PK. Detection of Leptomeningeal Disease Using Cell-Free DNA From Cerebrospinal Fluid. JAMA Netw Open. 2021 Aug 2;4(8):e2120040. doi: 10.1001/jamanetworkopen.2021.20040. PMID: 34369989; PMCID: PMC8353541.
